# Genomic insights into the pathogenicity of a ‘*Candidatus* Phytoplasma asteris’ associated with *Trema levigata* witches’ broom disease in China

**DOI:** 10.1186/s12870-025-07482-x

**Published:** 2025-10-28

**Authors:** Qiao Kai, Wan Qionglian, Li Xuemei, Wang Lianchun, Su Fan, Lei Jinfu, Shangguan Muzi, Li Mei, Shahzad Munir, Cai Hong

**Affiliations:** 1 Key Laboratory of Agro-Biodiversity and Pest Management of Education Ministry of China, Yunnan Agricultural University, Kunming, 650201 China; 2 School of Agronomy and Biological Science, Yuxi Normal University, Yuxi, 653100 China; 3 Plant Protection and Quarantine Station of Jiangcheng Hani and Yi Autonomous County, Puer, Yunnan 665900 China

**Keywords:** Phytoplasma, *Trema levigata* witches' broom disease, Genome assembly, Potential mobile units

## Abstract

**Background:**

Phytoplasma research encounters limitations due to the lack of availability of pure cultures of these microorganisms. In this study a culture-independent approach was employed to investigate the genome and pathogenic mechanisms of phytoplasma responsible for witches’ broom disease in *Trema levigata* (Yunnan province, China). The phytoplasma genome was assembled using Illumina sequencing data and the Phytoassembly pipeline based on mixed samples.

**Results:**

Nested PCR analysis identified a 16Sr group I, ‘*Candidatus* Phytoplasma asteris’ strain in *Trema levigate* showing witches’ broom disease. Comparative study between infected and healthy plants resulted in an 849.7 kb draft genome with 94.1% coverage, 27.6% GC content, encoding 1356 predicted genes, of which 587 were functionally annotated. Multilocus phylogenetic analysis showed that this phytoplasma is closely related to ‘*Ca.* P. asteris’. The genome possesses complete pathways for glycolysis and pyruvate metabolism pathways and harbors specific transporters (spermidine/putrescine, lysine), indicating dependency on host metabolites. Thirty-one putative secreted proteins, including TENGU and SAP05/54-like effectors were identified along with three potential mobile units carrying replication/recombination genes.

**Conclusions:**

This approach demonstrates the effectiveness of pathogen purification-free analysis for phytoplasma studies in naturally infected host species, thereby enhancing the understanding of phytoplasma-host interactions.

**Supplementary Information:**

The online version contains supplementary material available at 10.1186/s12870-025-07482-x.

## Introduction

Phytoplasmas are cell-wall-deficient, obligate prokaryotic pathogens that colonize plant phloem and insect vectors [1–3]. Their unique biology have historically constrained research progress [4]. Although these pathogens are associated with diseases in hundreds of globally important agricultural and forestry crops, persistent limitations of traditional methods have left key gaps in understanding phytoplasma genome structure, pathogenicity mechanisms, and evolutionary adaptation [5]. In the Yunnan Province, a recognized biodiversity hotspot in China, it was reported phytoplasma infections affecting five native tree species and seven commercial crops [6, 7]. However, genomic characterization of indigenous phytoplasma strains in this region remains scarce.

The unique biology of phytoplasmas creates inherent research challenges. Prolonged adaptation to nutrient-abundant host environments has resulted in the loss of essential metabolic pathways, including enzymes required for the tricarboxylic acid (TCA) cycle, pentose phosphate pathway, and fatty acid biosynthesis, making in vitro culture of pure strain not yet achieved [8–10]. Furthermore, uneven distribution within host tissues complicate enrichment of their genetic materials. Conventional techniques like cesium chloride density gradient centrifugation and pulsed-field gel electrophoresis (PFGE) have achieved partial genome isolation but are limited by high costs and technical complexity [11, 12]. Metagenomic approaches leveraging next-generation sequencing offer alternatives, although the lack of host reference genomes presents significant challenges [4]. Recent bioinformatic pipelines that exploit differences in sequencing coverage between host and pathogen in Illumina data have markedly improved genome assembly efficiency for phytoplasmas infecting non experimental host species [13].

Currently available phytoplasma genomes (>30 published) show significant reduction (500–900 kb) and often contain a number of Potential Mobile Units (PMUs) [14]. These transposase-associated gene clusters may promote genomic flexibility through recombination, potentially enhancing environmental adaptability [11]. Importantly, membrane-associated and secreted effector proteins (TENGU) are confirmed as possible key virulence determinants responsible for hallmark symptoms, including witches’ broom and stunting [3, 15, 16]. Considering the likely strain-specific differences in pathogenic mechanisms, expanding the genomic repository is essential to better elucidate evolutionary and virulence patterns.

*Trema levigata* (*Cannabaceae*), a pioneering tree species known for its rapid growth and ability to thrive in nutrient-poor soils, holds significant ecological and economic value. However, a witches’ broom disease was discovered in Xinping Yi and Dai Autonomous County, Yuxi City, Yunnan Province, China, causing considerable growth reduction and plant death. Initial phylogenetic analysis using 16 S rRNA gene has identified the associated pathogen as a ‘*Candidatus* Phytoplasma asteris’ strain (16SrI group); however, the absence of genomic data has hindered a full understanding of its disease mechanisms [17]. This study examines the *T. levigata* witches’ broom agent detected in Yunnan, China by using the high-throughput Illumina sequencing coupled with the Phytoassembly bioinformatics pipeline. This was applied for the first time to this disease to assemble the phytoplasma genome directly from mixed host-pathogen samples. This culture-independent and host-reference-free approach simultaneously yields genomic data for both the host and the pathogen, providing an efficient and cost-effective strategy for phytoplasma research. Through comparative genomics and functional annotation, it was elucidated *T. levigata* witches’ broom phytoplasma adaptive traits and potential virulence factors were identified, thereby establishing a molecular basis for disease management while offering a methodological framework for genomic studies of uncultured phytoplasmas.

## Materials and methods

**Fig. 1:**
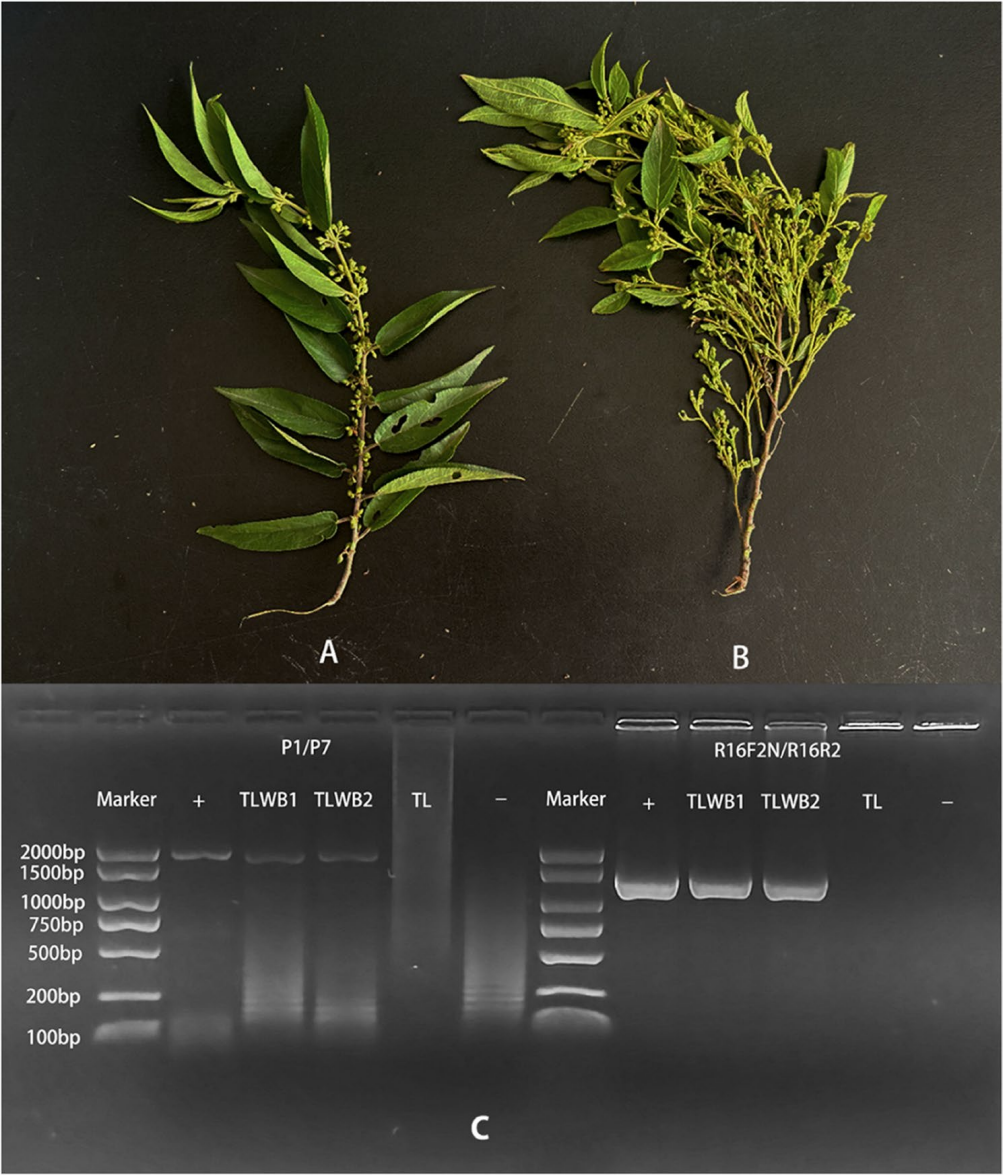
Comparative morphology of healthy and witches’-broom symptomatic *Trema levigata* plants and Nested PCR detection. **A**: Healthy T. levigata; **B**: T. levigata exhibiting witches'-broom symptoms **C**: Nested PCR detection of phytoplasma 16S rRNA gene (Lane M: 2000 bp DNA marker; + : Positive control (Camptotheca acuminata witches'-broom phytoplasma GenBank: GCA_041276565.1); TLWB1/TLWB2: Symptomatic T. levigata samples 1/2; TL: Asymptomatic T. levigata; -: Negative control (NTC))；Suppl. Fig. 1: 1% TBE Agarose gel of PCR-amplified phytoplasma DNA.，Suppl. Fig. 2: Field symptoms of Trema levigata witches' broom

Wild-growing symptomatic *T. levigata* samples from plants exhibiting witches’ broom disease (Fig. 1B) were collected from Pingdian Township, Xinping Yi and Dai Autonomous County, Yuxi City, Yunnan Province, China (Coordinates: 101.843422°E, 24.021594°N). Healthy *T. levigata* samples (Fig. 1A) were collected from Huanian Town, Eshan Yi Autonomous County, Yuxi City, Yunnan Province (Coordinates: 102.211513°E, 24.087931°N). All plant samples were identified as *T. levigata* by Dr. Qionglian Wan based on the Flora of China, and confirmed by comparison with voucher specimens at the Herbarium of the Institute of Botany, Chinese Academy of Sciences, Beijing city, China (voucher number: 01847045).

### Nucleic acid extraction and nested PCR Detection

Total genomic DNA was extracted from 3 g of fresh leaf tissue following grinding in liquid nitrogen, using the Omega Plant DNA Extraction Kit (Omega, Georgia, US) according to the manufacturer’s instructions. Nested PCR amplification targeting the phytoplasma 16 S rRNA gene was performed using universal primer pairs P1/P7 and R16F2n/R16R2 (nested PCR reaction) [18–21]. A laboratory-maintained *Camptotheca acuminata* phytoplasma sample (GenBank: GCA_041276565.1) was used as the positive control, and DEPC-treated water served as the negative control. PCR amplification was conducted using Phanta^®^ Max Super-Fidelity DNA Polymerase (Vazyme Biotech, Nanjing, China). The 25 µL reaction mixture contained: 12.5 µL of 2× Phanta Max Buffer, 0.5 µL of dNTP Mix (10 mmol·L⁻¹ each), 1 µL each of forward and reverse primers (10 µmol·L⁻¹), 0.5 µL of Phanta^®^ Max DNA Polymerase, 0.5 µg of DNA template, and an appropriate volume of DEPC-treated sterile ultrapure water to make a final volume of 25 µL. PCR products were resolved on a 1% TBE agarose gel, and amplicons were purified and subjected to Sanger sequencing (Tsingke Biotechnology, Beijing, China).

### Genome sequencing and data processing

Leaf midribs were collected from three individual witches’ broom-diseased plants (positive biological replicates) and one healthy plant (negative control). Leaf midribs from PCR-positive symptomatic samples and healthy controls were dissected, flash-frozen in liquid nitrogen, and stored at −80 °C. Total genomic DNA was extracted from 0.5 g sample aliquots. Sequencing libraries were constructed and subjected to whole-genome sequencing (WGS) on an Illumina NovaSeq X Plus platform (Personalbio, Shanghai, China) with a paired-end (PE) 2 × 150 bp read configuration. The sequencing depth was 10 Gb per sample. Raw sequencing reads were quality-controlled and adapter-trimmed using FastQC (Version 0.12.1). De novo genome assembly followed the Phytoassembly pipeline (Version 0.9.2) described by Cesare et al. [13]. following sequencing and preliminary assembly of all samples, the dataset demonstrating the most robust assembly quality—based on comprehensive evaluation including contiguity (e.g., N50) and completeness—was selected for subsequent in-depth analysis.

### Genome analysis

The draft phytoplasma genome was annotated using the RAST server [22](https://rast.nmpdr.org/, accessed April 1, 2025). To identify putative secreted proteins, the complete set of predicted protein sequences was analyzed as follows: SignalP v6.0 [23](https://services.healthtech.dtu.dk/services/SignalP-6.0/ accessed April 1, 2025) was used to predict signal peptides. Given that phytoplasmas are divergent Gram-positive bacteria, we selected the ‘Other’ organism group for prediction. To maximize prediction accuracy and detail, we used the ‘Slow’ model with ‘Long’ output format. This analysis was performed on the full-length protein sequences; all input sequences were greater than 10 amino acids in length and thus within the reliable prediction range of the tool. Mature protein sequences (i.e., sequences after the predicted signal peptide cleavage site) were subsequently analyzed with TMHMM v2.0 [24] to identify transmembrane domains. Proteins without predicted transmembrane domains were manually inspected to exclude those with well-characterized functions (*e.g*., ABC transporters); remaining candidates were annotated as putative secreted proteins.

The MOTIF search tool(https://www.genome.jp/tools/motif/MOTIF.html accessed April 1, 2025) identified protein domains associated with effectors and Potential Mobile Units (PMUs) [25]. proksee facilitated genome visualization༈https://proksee.ca/ accessed April 1, 2025༉ [26]. Orthologous single-copy sequences were identified using OrthoFinder [27]༈ver 2.5.5༉. Multiple sequence alignment employed MUSCLE༈ver 5.3༉ [28], with conserved blocks extracted using Gblocks༈ver 0.91b༉ [29]. Concatenated alignments were analyzed with ProtTest3 (ver 3.4.2) [30]to determine optimal substitution models. Maximum-likelihood phylogenetic trees were constructed using MEGA (ver 12) [31]. Metabolic pathway reconstruction utilized BlastKOALA [32]༈https://www.kegg.jp/blastkoala/ accessed April 1, 2025༉ against the KEGG database. Functional annotation was performed with eggNOG-mapper [33]༈http://eggnog-mapper.embl.de/ accessed April 1, 2025༉.

## Results

On July 22, 2024, symptoms of witches’ broom were observed on *T. levigata* plants in Xinping County (Supplemental Fig. 2). Branches exhibiting witches’ broom symptoms were collected and processed in the laboratory, where nested PCR detection confirmed phytoplasma presence (Fig. 1C). Subsequently, both negative (healthy) and positive (infected) plant samples were submitted to Shanghai Personal Biotechnology Co., Ltd. (China) for genomic DNA library preparation and whole-genome sequencing (WGS) on an Illumina NovaSeq X Plus platform with a paired-end (PE) 2 × 150 bp read configuration (150 bp read length), achieving a sequencing depth of 10 Gb per sample. Raw data were deposited in NCBI under accession numbers SRR33445509 and SRR33445508. After quality control using FastQC, the phytoassembly pipeline was employed for genome assembly. The assembled genome size of the negative plant was 364 Mb, which served as the reference genome for subsequent analyses. The comparison between the positive plant genome and negative reference genome resulted in 1.4 Gb of non-matching reads, which were further filtered to obtain an 836 kb phytoplasma genome.

### General features of the T. levigata and phytoplasma genomes

**Fig. 2:**
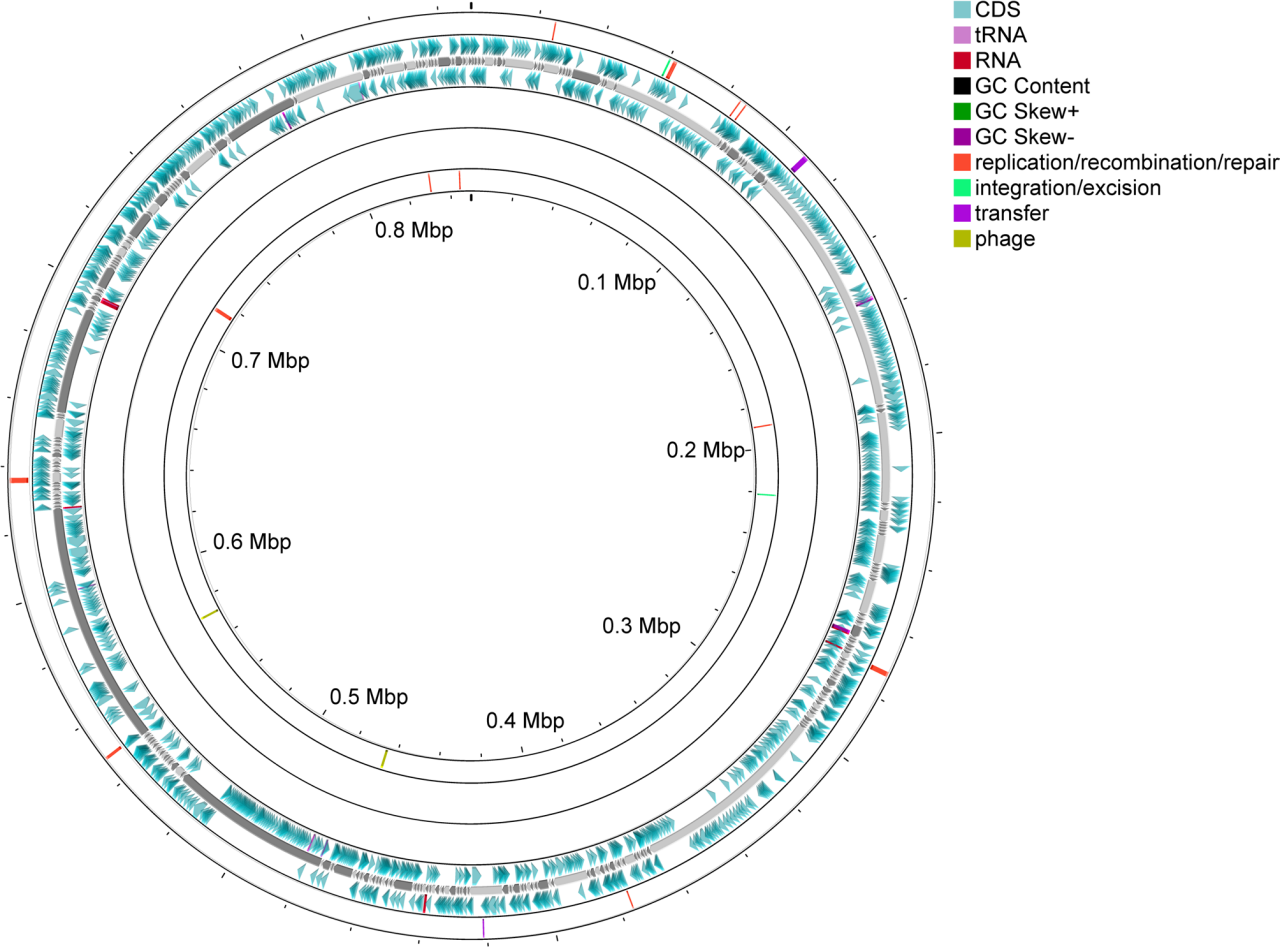
Draft genome map of the *Trema levigata* witches’ broom phytoplasma. The innermost and outermost rings represent mobileOG annotations for the negative and positive strands, respectively: red indicates replication/recombination/repair, yellow-green represents integration/excision, bright purple denotes transfer, and light green indicates phage. The second ring from the inside displays GC Skew. The third ring shows GC content. The fourth and sixth rings correspond to annotated features on the negative and positive strands, with blue representing CDS, dark red indicating RNA, and light purple denoting tRNA. The fifth ring illustrates the draft genome framework.This figure was generated using Proksee (accessed April 1, 2025; https://proksee.ca/)

The positive sample, containing 9.8 Gb of raw sequencing data, provided 11,652× coverage of the *T. levigata* witches’ broom phytoplasma genome, which was assembled into 380 contigs with a total size of 849,708 bp (Fig. 2; NCBI BioSample number: SAMN48173560). BUSCO assessment revealed a draft assembly completeness of 94.1%, with a GC content of 27.6%, an N50 of 23,241 bp, and an L50 of 9. RAST annotation identified 1,356 coding sequences (CDSs), of which 587 had defined functions and 769 were hypothetical proteins (Supplementary Table 1). Additionally, 34 ribosomal sequences were annotated (16 rRNAs and 28 tRNAs).

The negative sample, with 9.6 Gb of raw sequencing data, provided 26× coverage of the *T. levigata* genome, which was assembled into 325,004 contigs with a total size of 362,568,904 bp (NCBI BioSample number: SAMN48908987). BUSCO assessment indicated a genome assembly completeness of 92%, with 7.3% of genes partially covered and 0.7% missing. The GC content was 33.9%, and the N50 was 13,457 bp. Augustus annotation predicted a total of 62,862 CDSs.

### Phylogenetic analysis

**Fig. 3:**
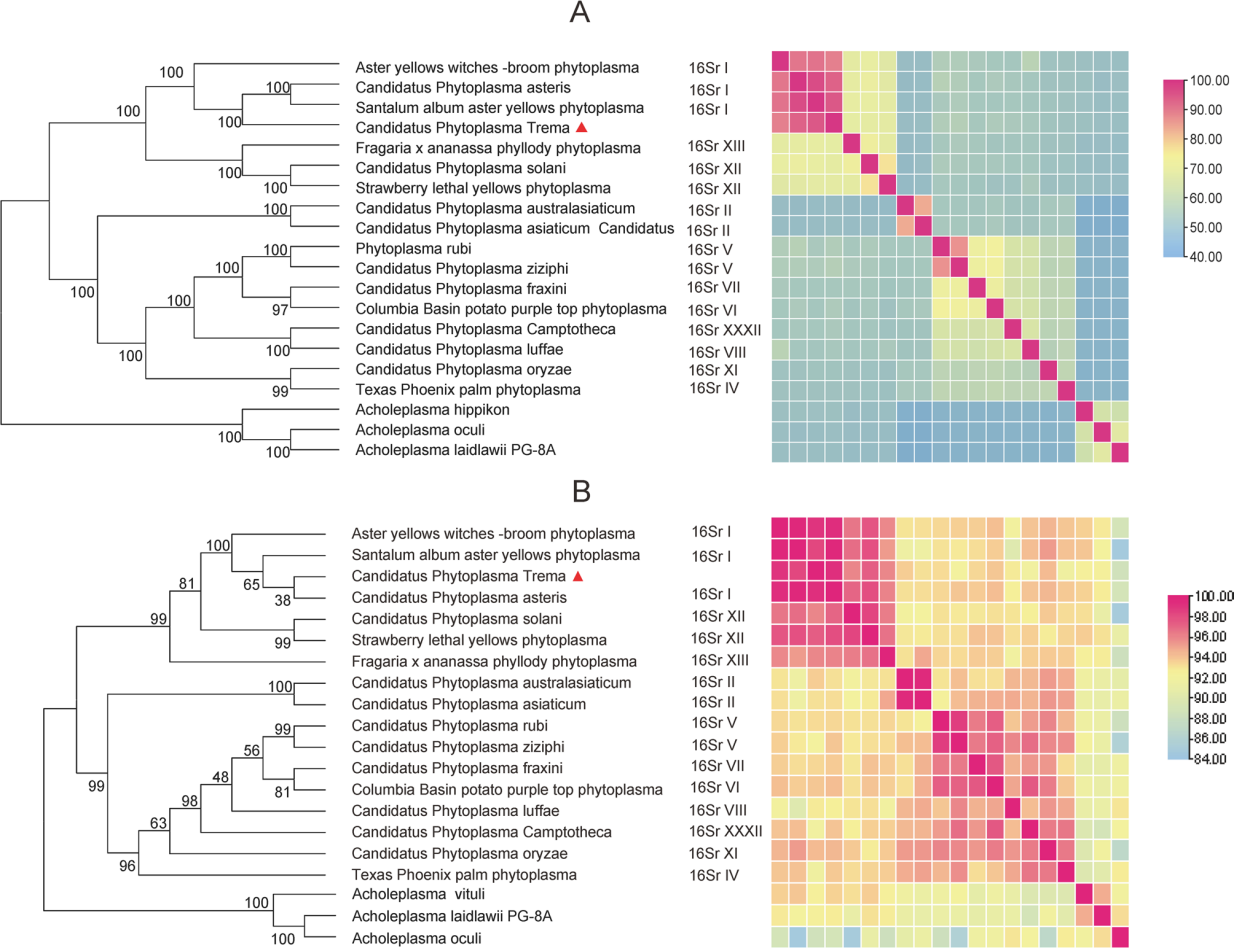
Molecular genetic analyses. **A**: Phylogenetic tree based on 139 homologous single-copy protein sequences; **B**: Phylogenetic tree based on the 16 S rRNA gene.The *Acholeplasma* species were included as outgroups to root the tree. Numbers on branches indicate bootstrap support values (based on 1000 replicates). The numbers at the nodes represent the number of substitutions per site. The heatmaps on the right represent pairwise nucleotide identity (B) and amino acid identity (A), respectively.In (A), Average Amino Acid Identity (AAI) values are color-coded as follows: >80% (red), > 60% (yellow), and < 60% (blue).In (B), Average Nucleotide Identity (ANI) are indicated as: >94% (red), > 88% (yellow), and < 88% (blue)

Phylogenetic analyses were performed using 16 S rRNA sequences and 12 orthologous single-copy genes, with extended analysis of 139 orthologous genes provided enhanced taxonomic resolution and stronger nodal support. The newly identified *T. levigata* witches’ broom phytoplasma exhibited 95.38% ANI and 99.03% 16 S rRNA sequence identity with ‘*Ca*. *P. asteris’*(GeenBank: GCA 038505995.1), forming a well-supported monophyletic clade within the 16SrI group that included *Santalum album* aster yellows phytoplasma (GeenBank: GCA 018283495.1), Aster yellows witches’-broom phytoplasma (GeenBank: GCA 000012225.1), and ‘*Ca*. *P. asteris’* (GeenBank: GCA 038505995.1) (Fig. 3, Supplementary Table 2). These results corroborate the previous findings regarding the phylogenetic positioning of this phytoplasma lineage [17].

### Functional classification and analysis of phytoplasma genes

COG annotation of the *T. levigata* witches’ broom phytoplasma genome identified 697 functionally annotated genes. The top ten functional categories were: Replication, recombination and repair (L); Posttranslational modification, protein turnover, chaperones (J); Chaperones and stress response (O); Transcription (K); Function unknown (S); Cell cycle control, division and chromosome partitioning (D); Nucleotide transport and metabolism (F); Inorganic ion transport and metabolism (P); Amino acid transport and metabolism (E); and Defense mechanisms (V) (Supplementary Tables 3 and Supplementary Fig. 1). Further annotation revealed that the 142 genes in category L primarily encoded DnaB, YqaJ, and AAA + family proteins. Category J contained 122 genes, dominated by ribosomal proteins and tRNA-related elements. The 90 genes in category O were predominantly AAA family chaperones. Category S comprised 53 genes mainly belonging to DUF families, methyltransferases, and PmbA_ family proteins. Category K included 50 genes encoding sigma factors, while category D featured 47 genes associated with DUF and CheB families. Category F contained 49 genes principally involved in thymidylate and flavodoxin functions. Category P encompassed 38 genes encoding amino acid transport, ABC transporters, lipoproteins, and cation ATPases. Category E consisted of 23 genes encoding peptidases, MCP signal proteins, and asparagine synthases. Category V comprised 15 genes encoding ABC membrane transporters, MatE multidrug efflux systems, and HsdM restriction-modification components.

**Fig. 4:**
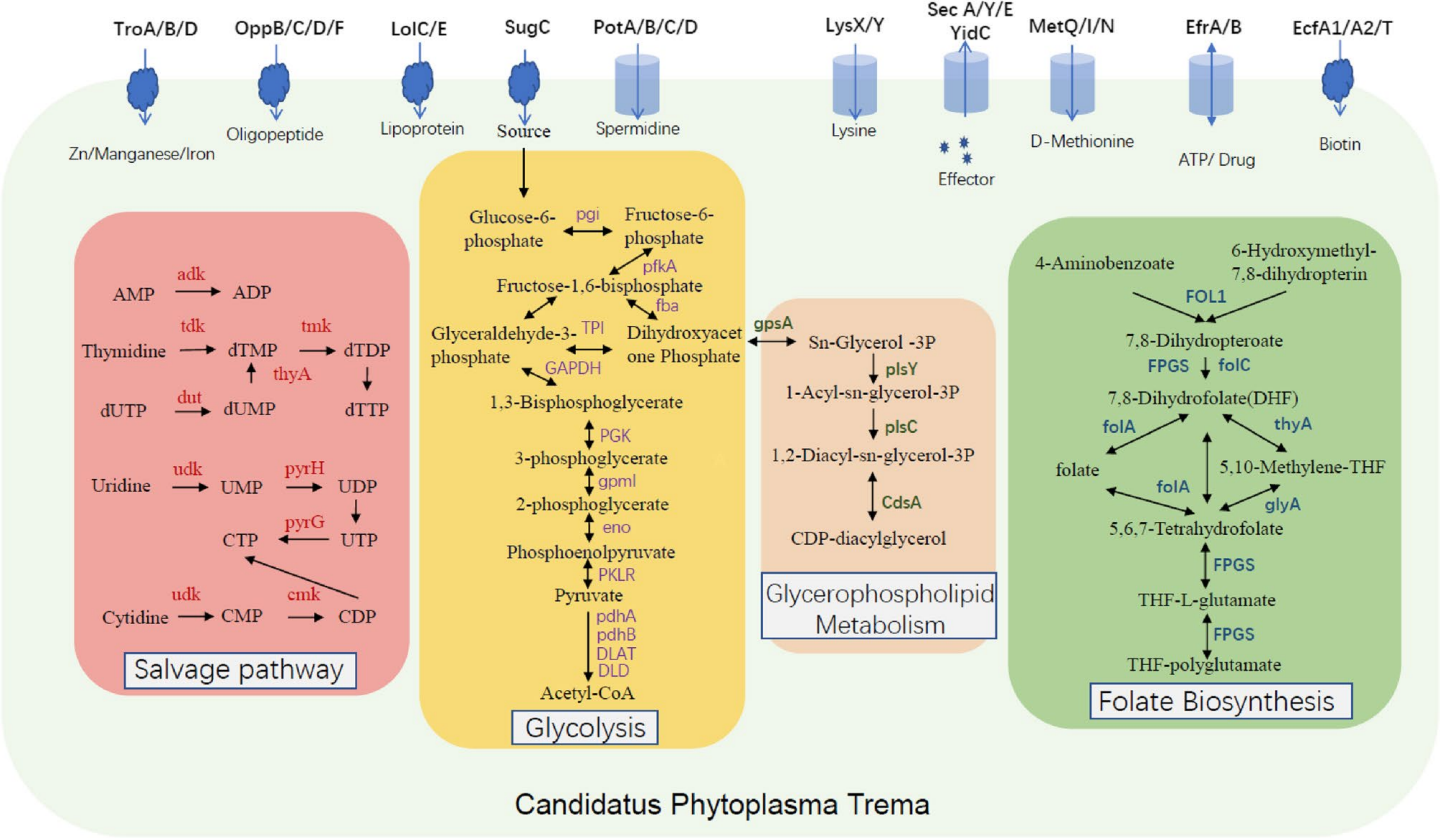
An overview of the metabolic pathways in *Trema levigata* witches’ broom phytoplasma. The functional genome, predicted through KEGG analysis, reveals key metabolic pathways and transport proteins. Genes associated with the salvage pathway are indicated in red; genes involved in the glycolysis pathway are marked in purple. Trema levigata witches’ broom phytoplasma can fully utilize glucose-6-phosphate to generate acetyl-CoA. Genes related to the glycerophospholipid metabolism pathway are shown in dark green; genes involved in the folate biosynthesis pathway are highlighted in blue. A variety of transporter system genes—including TroA/B/D, OppB/C/D/F, LolC/E, SugC, EcfA1/A2/T, PotA/B/C/D, LysX/Y, MetQ/I/N, SecA/Y/E, YidC, EfrA/B, and EcfA1/A2/T—are labeled on the membrane

KEGG annotation identified 573 genes, with the top five enriched pathways being Transporters, DNA repair and recombination proteins, Ribosome, Prokaryotic defense systems, and Transfer RNA biogenesis. Metabolic analysis revealed that *T. levigata* witches’ broom phytoplasma possesses a complete glycolytic core module for three-carbon compounds, pyruvate oxidation capability (converting pyruvate to acetyl-CoA via pyruvate dehydrogenase complex genes pdhA/B, DLAT, and DLD), phosphate acetyltransferase-acetate kinase pathway (acetyl-CoA to acetate conversion), and phosphatidylethanolamine (PE) biosynthesis (PA to PS to PE conversion). Reconstruction of its metabolic network (Fig. 4) confirmed retention of a complete glycolytic pathway from glucose-6-phosphate to pyruvate, with all key enzyme genes identified (pgi, pfkA, fba, TPI, gapA, pgk, gpmI, eno, pyk). Notably, it exhibited unique membrane lipid synthesis capabilities, including Sn-glycerol-3-phosphate production via gpsA-encoded dehydrogenase, CDP-diacylglycerol synthesis by CdsA, and a 1-acyl-sn-glycerol-3-phosphate pathway mediated by plsY and plsC. Complete nucleotide salvage pathways were identified, encompassing thymidylate synthesis, deoxyuridine triphosphate conversion (dut, tdk, tmk), and pyrimidine nucleotide interconversion networks (pyrH, pyrG, cmk), potentially supporting its high-frequency genomic recombination. Folate metabolism showed parasitic adaptation traits: retention of dihydrofolate reductase (folA) and dihydropteroate synthase (folP), but loss of *de novo* synthesis, indicating dependence on exogenous 4-aminobenzoate and pterin precursors (Fig. 4, Supplementary Table 3).

Strikingly, the metabolic network displayed high host dependency, evidenced by complete transport systems for spermidine/putrescine, lysine, and D-methionine; glutamine transporters GlnH and GlnP; bacitracin transporter ATP-binding protein BceA; ABC transporters EfrA/B; D-methionine permeases MetI/MetN; glutathione permease GsiD; EcfT/A1/A2 components; and ABCB subfamily transporters EfrA/B. Additionally, genes encoding the Sec-SRY secretion pathway—including *sec*A (ATPase), *sec*Y (channel pore), *sec*E (stabilizing subunit), and *yid*C (membrane integrase)—were identified, suggesting adaptive support for host exploitation through diverse transporter mechanisms.

### Effector analysis and identification of potential mobile Units

In the genome of the *T. levigata* witches’ broom phytoplasma, 31 putative secreted effector proteins were identified. Among these, three mature proteins were classified as small peptides (< 10 amino acids) following the removal of their signal peptides. These were classified as secreted based on a high-confidence prediction of an N-terminal signal peptide in their precursor sequences, followed by the absence of transmembrane domains in the mature protein. Annotation revealed eight unique putative effectors, while the remaining 20 showed homology to effector proteins in other phytoplasma strains. Five were previously characterized effectors: TENGU, SAP05, SAP06/48-like, and SAP54(Supplementary Table 4).

**Fig. 5:**
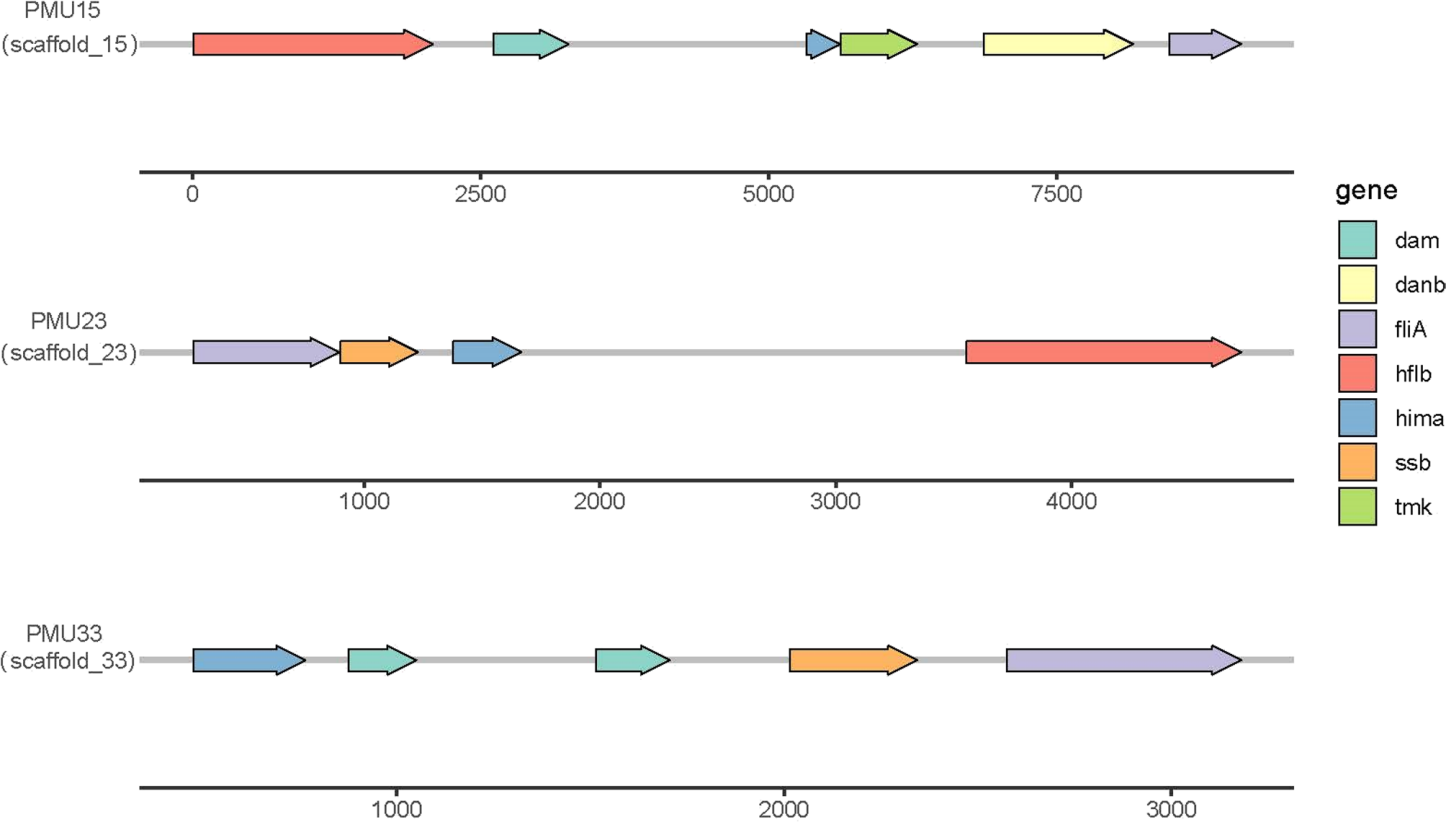
Schematic representation of potential mobile unite. Three potential mobile units (PMUs) were identified and designated as PMU15, PMU33, and PMU23. The assembly scaffold corresponding to each PMU is indicated in parentheses below its name. Numerical values on the horizontal axis denote the length of each PMU in base pairs (bp). Arrows of varying colors represent distinct genetic or functional components. A legend elucidating these components is provided on the right side of the figure

Motif analysis identified three putative Potential Mobile Units (PMUs) ranging from 2 to 9 kb (Fig. 5 and Supplementary Table 5). Functional annotation of coding sequences within these regions showed conserved genes across all PMUs, including ATP-dependent Zn protease, DNA-binding protein HU, DNA-directed RNA polymerase specialized sigma subunit, and site-specific DNA methylase. PMU15 uniquely encoded a thymidylate kinase-like protein; PMU23 contained a single-stranded DNA-binding protein; and PMU33 featured a replicative DNA helicase-like protein.

To investigate potential horizontal gene transfer (HGT) during long-term host-phytoplasma interactions, one candidate HGT sequence (E-value: 3.86E-75) was identified with homology to an aspartate aminotransferase gene, shared between the *T. levigata* and phytoplasma genomes. This sequence on phytoplasma scaffold_6 comprised three tandem asparagine synthetase genes. While the region also contained TRA5 and fliA annotations, no other canonical PMU marker genes were detected.

## Discussion

Phytoplasmas are intracellular parasitic bacteria characterized by unique metabolic features and genetic diversity resulting from their high dependence on host organisms. Through sequencing and analysis of the *T. levigata* witches’ broom phytoplasma genome, this study elucidates its metabolic network, transporter systems, secreted proteins, and potential mobile units (PMUs), providing insights into its pathogenic mechanisms and evolutionary adaptations.

### Streamlined methodology for phytoplasma genome assembly

The inability to date to establish reliable in vitro culture methods for phytoplasmas, discovered in 1967, continues to be a key limiting factor, impeding the procurement of high-quality DNA and advancing pathogenicity studies [34–36]. Traditionally, researchers extracted phytoplasma DNA from infected plant tissues, a challenging task due to the pathogen’s low and variable abundance (particularly in woody hosts), which necessitated complex enrichment and purification techniques such as CsCl equilibrium buoyant density gradient centrifugation (requiring bisbenzimide dyes) or pulsed-field gel electrophoresis (PFGE) for whole-chromosome isolation [37, 38].

Early genome sequencing efforts focused on ‘*Ca*. P. asteris’ strain OY-M [39] and ‘*Ca.* P. mali’ AT [38, 40]. However, the pronounced base-composition bias in the AT strain introduced specific assembly difficulties. To address this, long-read sequencing technologies and metagenomic approaches were adopted [4, 41]. The latter employs bioinformatic strategies to efficiently filter pathogen sequences from randomly sequenced DNA libraries of diseased plant samples. Notably, when constructing draft phytoplasma genomes using next-generation sequencing (NGS), the primary technical bottleneck lies in accurately identifying and separating pathogen genomic sequences from vast sequencing datasets.

Cesare Polano’s team innovatively developed the Phytoassembly bioinformatics pipeline to overcome this challenge. Built upon the IDBA-UD assembler, this workflow is optimized explicitly for mixed samples with uneven sequencing coverage, and its automated design enables rapid analysis even by researchers without specialized genomic expertise [13]. In this study, high-throughput Illumina sequencing of healthy and infected plants was performed, and the resulting data processed through this pipeline successfully generated a draft genome of *T. levigata* witches’ broom phytoplasma with 94.1% completeness. This methodological advancement markedly reduces the complexity of phytoplasma genome research and offers an essential groundwork for future functional investigations.

### Phytoplasma genomic characteristics and structural analysis

Phytoplasma genomes generally demonstrate reductive evolution, with sizes ranging from 400 to 1,000 kbp, similar to other plant obligate pathogens such as ‘*Ca* Liberibacter asiaticus’ [42]. The assembled *T. levigata* witches’ broom phytoplasma genome (856 kbp) falls within this range. However, its assembly, derived from Illumina short-read data, consisted of 390 contigs, showing a much higher level of fragmentation than previously documented phytoplasma genomes. This discrepancy may be due to: (1) the absence of a reference genome for the host plant *T. levigata*, which makes subtracting host DNA more difficult; and (2) the high complexity of non-matching reads from infected samples when reference genomes from healthy plants are used.

Notably, phytoplasma genomes possess a unique chromosomal organization with unclear evolutionary origins. Studies suggest viral sequences may influence genomic architecture; Wei et al. demonstrated Caudovirales phages critically shaped ‘*Ca*. P. asteris’ genome [43]. Here, MobileOG-db annotation identified three phage-related sequences, though none localized to Potential Mobile Units (PMUs)—potentially due to PMU detection challenges from high fragmentation. Intriguingly, PMU23 and PMU33 harbored abundant replication/recombination/repair elements, implicating these regions in genomic plasticity.

COG annotation identified 697 genes, comparable to ‘*Ca*. P. ziziphin’ [44] but exceeding other sequenced strains. This discrepancy may arise from: (1) gene duplication artifacts from tandem repeats; (2) pseudogene interference; or (3) strain-specific gene expansion. These findings highlight limitations of short-read assemblies, suggesting long-read sequencing could enhance completeness and annotation accuracy for deeper insights into phytoplasma evolution and pathogenesis.

### Phylogeny and metabolic adaptations

Phytoplasma taxonomy adheres to the guidelines established by the IRPCM Phytoplasma/Spiroplasma Working Team, which require a minimum of 98.65% 16 S rRNA sequence identity and 95% Average Nucleotide Identity (ANI), as well as multilocus sequence analysis (MLSA) [45, 46]. *T. levigata* witches’ broom phytoplasma showed 99.25% 16 S rRNA identity and 95.38% ANI with ‘*Ca.* P. asteris’ (GeenBank: GCA 038505995.1). Phylogenetic analysis based on orthologous single-copy genes demonstrated its closest relationship with *Santalum album* aster yellows phytoplasma, showing slight divergence from trees constructed using 16 S rRNA data. Nonetheless, all strains with high homology belong to the 16SrI group, which aligns with previous MLSA findings. As a ubiquitous group globally, 16SrI phytoplasmas infect *T. levigata*, a pioneer plant species widely distributed across Yunnan Province, China. During surveys conducted in 2022, witches’ broom symptoms were observed on *T. levigata* at three sites—namely Shangri-La and Yuxi—with phytoplasma presence confirmed. Further investigation is required to identify the insect vectors involved and to understand the mechanisms of pathogenesis. As obligate pathogenic prokaryotes, phytoplasmas exhibit reductive genomics and metabolic remodeling. Adapted to nutrient-rich phloem environments, they lack core metabolic pathways (e.g., TCA cycle, complete oxidative phosphorylation), instead evolving diverse energy-acquisition strategies: some strains (e.g., ‘*Ca*. P. asteris’ strain OY-W) amplify glycolysis via gene duplication, while others (e.g., ‘*Ca*. P. mali’) lose glycolytic genes and rely on alternative pyruvate synthesis [39]. It shares a similar glucose to pyruvate conversion pathway with ‘Ca. P. rubi’, which lacks the genes for inorganic pyrophosphatase and F1Fo-ATP synthase, thus rendering it incapable of establishing a complete electron transport chain. Notably, phytoplasmas maintain membrane potential, likely through transmembrane electrochemical gradients established by P-type ATPases (orthologous to eukaryotic Na^+^/K^+^- or H^+^/K^+^-ATPases). This unique energy-conversion mechanism may represent a key adaptation after the loss of standard oxidative phosphorylation. Such interspecific metabolic variations reflect adaptations to host environments, and their streamlined yet specialized energy networks provide critical insights into pathogenicity mechanisms.

### Secretory pathways and effector mechanisms

Due to the lack of essential metabolic enzymes, phytoplasmas have developed various transporter systems to effectively extract vital nutrientsincluding sugars, amino acids, oligopeptides, and inorganic ionsfrom their hosts, demonstrating high metabolic dependence [47]. Studies indicate that phytoplasmas primarily utilize two secretion systems for parasitism: YidC mediates membrane protein integration, while the Sec system facilitates protein translocation and secretion into the host cytoplasm [48]. The Sec protein translocation system, essential for bacterial viability [49, 50], is well-characterized in *Escherichia coli*. Its core components, SecA, SecY, and SecE, are indispensable for translocation activity and cell survival; remarkably, these three proteins alone are sufficient to reconstitute translocation in vitro [51].

Conservation of the Sec system in phytoplasmas is well-established. Genes encoding SecA, SecY, and SecE were identified in ‘*Ca*. P. asteris’ strain OY-M [52], with SecA expression confirmed in infected plants [53]. These genes have also been reported in several other phytoplasma genomes [38, 54, 55], and *secY* has been cloned from multiple strains. Collectively, this evidence indicates that functional Sec systems are ubiquitous in phytoplasmas. In *T. levigata* witches’ broom phytoplasma, genes encoding the complete core Sec machinery (*secA*, *secY*, *SecE*) and *yidC* were identified, indicating the presence of the typical phytoplasma secretion mechanisms.

Regarding ATP-binding cassette (ABC) transporters, *T. levigata* witches’ broom phytoplasma exhibits distinct metabolic traits. The retention of a complete set of glycolysis genes correlates with a reduced number of transporters. Only intact spermidine/putrescine, lysine, and D-methionine transport systems were identified.

Polyamines (PAs), nitrogen-rich compounds containing multiple amine groups, play vital roles in plant development and stress responses [56]. Pathogen infection significantly induces the expression of PA metabolism genes [57], and disease-resistant cultivars accumulate higher levels of PA metabolites under stress [58]. The retention of PA transport channels in *T. levigata* witches’ broom phytoplasma may reflect a specialized strategy for nitrogen acquisition. Exogenous PAs could be a critical nitrogen source without complete amino acid biosynthesis pathways. Furthermore, putrescine and lysine are known to mitigate intracellular toxin accumulation [59]. This suggests that the methionine/lysine transporters facilitate nitrogen acquisition and contribute to neutralizing phytochemical toxicity. These insights advance the understanding of the nutritional adaptations underpinning host-phytoplasma coevolution.

### Phytoplasma secreted proteins

Genomic analyses indicate phytoplasmas typically encode >10 secreted proteins, some characterized as effectors [60, 61]. Functionally, these effectors fall into two classes: (1) those inducing witches’ broom, leaf curling, abscission, dwarfism, and sterility by modulating plant defenses or disrupting cellular structures; and (2) those regulating plant-insect interactions to facilitate vector feeding and reproduction.

This study identified multiple known effectors in *T. levigata* witches’ broom phytoplasma, including TENGU, SAP05, SAP54, and SAP06/48-like. Crucially, 16SrI-group effectors (e.g., SAP11, SAP05, TENGU, SAP54) have defined mechanisms: SAP11 destabilizes TCP transcription factors to promote axillary branching [16, 62]; SAP05 degrades SPL/GATA factors via ubiquitin-independent proteasomal pathways, causing leaf malformation and delayed flowering [41, 63]; SAP54 degrades MADS-box factors via RAD23 interaction, inducing floral reversion [64]. Notably, identical effectors may function differentially across host systems. For instance, SAP11, SWP1, SJP1, and SJP2 require N-terminal nuclear localization signals and C-terminal coiled-coil domains to regulate TCP stability [65], while SAP54 from paulownia witches’ broom phytoplasma induces branching, whereas its AY-WB homolog causes leaf splitting [66, 67]. This functional diversity reflects adaptive strategies shaped by host coevolution. Additionally, five sequence-variable mosaic homologs of uncharacterized secreted proteins were identified. These potential novel host-interaction factors represent targets for elucidating phytoplasma pathogenesis. The witches’ broom symptoms in *T. levigata* likely arise from synergistic actions of these effectors.

### Potential mobile units (PMUs)

Based on Bai et al.’s eight core genes (*tra5*, *dnaB*, *dnaG*, *tmk*, *hflB*, *himA*, *ssb*, *rpoD*), PMUs were analyzed [54]. Three PMU-like regions were identified in *T. levigata* witches’ broom phytoplasma, exhibiting characteristic organizational heterogeneity and gene rearrangements, aligning with their established role in genomic diversification and horizontal transfer. Contrary to the findings of Huang et al. [41], no known effectors (e.g., SAP11/SAP09) were detected in these PMUs—possibly due to assembly fragmentation. The presence of numerous hypothetical proteins within these canonical mobile genetic elements strongly suggests that they are functional components. Their co-localization with replication genes implies they may be mobilized as a unit, potentially facilitating the rapid evolution of virulence by disseminating these unknown genes across the phytoplasma population. Intriguingly, these regions were enriched in protein-synthesis enzymes (e.g., ATP-dependent Zn protease, methionyl-tRNA synthetase) co-localized with DNA replication/translation genes (*dnaB*, *ssb*), potentially enabling rapid protein synthesis during host shifts. FtsHs (encoded by the *hflB* gene) are membrane-associated ATP-dependent Zn proteases that degrade certain membrane proteins which have not been assembled into complexes. In ‘*Candidatus* Phytoplasma ziziphi’, the ATP-dependent Zn protease is significantly upregulated during infection. This upregulation may be associated with the functional role of effector proteins in the infection process [68].

A putatively horizontally transferred gene encoding asparagine synthetase an enzyme essential for nitrogen metabolism was identified on scaffold_6. This finding aligns with the previously observed abundance of amino acid transporters, highlighting the phytoplasma’s reliance on external nitrogen sources. Supporting this, Wei et al. documented an upregulation of asparagine synthetase in *Solanum lycopersicum* during phytoplasma infection, thereby underscoring the enzymes significance and its role in pathogenesis [69]. These findings suggest that horizontal transfer of asparagine synthetase may constitute an adaptive metabolic strategy for pathogenicity.

###  Limitations of the study

Although this study provides important insights into the genome of the phytoplasma associated with *T. levigata* witches’ broom disease, several limitations should be noted. The analysis relied on only three technical replicates and one negative control, which may limit the generalizability of the results given the sparse natural distribution of infected plants. The genome assembly is highly fragmented (380 contigs, N50 = 23,241 bp), due to the use of Illumina short-read sequencing and the lack of a reference genome for the host plant, complicating the separation of phytoplasma-derived sequences. This fragmentation increases the risk of missing genomic regions, incomplete gene annotation, and failure to detect structurally complex elements such as PMUs or effector genes. To enhance assembly continuity, future work may focus on enriching phytoplasma DNA followed by long-read sequencing using platforms such as PacBio or Oxford Nanopore.

## Conclusions

Through systematic genomic analysis of a phytoplasma associated with *T. levigata* witches’ broom disease in Yunnan, China, this study generated a high-quality draft genome (849.7 kb, 94.1% completeness) of a novel 16SrI group ‘*Ca*. P. asteris’ strain (provisionally designated *T. levigata* witches’ broom phytoplasma). The genome exhibits classic reductive features of phytoplasmas while uniquely retaining complete glycolysis and pyruvate metabolism pathways. It additionally possesses specialized polyamine/amino acid transporter systems, suggesting an adaptive strategy involving hijacking of host nitrogen metabolism. The identification of 31 secreted proteins, three potential mobile units, and a putative horizontal gene transfer locus encoding asparagine synthetase provides new insights into the pathogenesis of phytoplasma. Methodologically, the innovative use of the Phytoassembly pipeline enabled genome resolution without pathogen purification. This establishes a molecular foundation for controlling *T. levigata* witches broom disease and offers a methodological framework for studying other phytoplasmas. Future research should focus on the functional validation of effectors and investigation of host-interaction mechanisms mediated by horizontally acquired genes to elucidate phytoplasma-host co-evolutionary dynamics.

## Supplementary Information

Supplementary Material 1. Additional file S1: Supplementary Table S1. Supplementary Table 1: RAST annotation of the genome of Trema levigate witches’-broom phytoplasma; Supplementary Table 2 Phylogenetic Analysis data; Supplementary Table 3 Results of eggnog annotation of CDS of Trema levigate witches’-broom phytoplasma; Supplementary Table 4 potential secretory proteins; Supplementary Table 5 Result of MotifFinder PMU

Supplementary Material 2. Additional file S2: Supplementary figure S2. Supplemental Fig. 1: Original agarose gel image of PCR detection for phytoplasma; Supplemental Fig. 2: Field symptoms of witches’ broom disease on Trema levigata

## Data Availability

Data supporting the findings of this study are available within the paper and its Supplementary Information files. A reporting summary for this article is also provided in the Supplementary Information. The datasets and plant materials generated and analysed during the current study are available from the corresponding author upon reasonable request. The raw sequencing data have been deposited in the NCBI Sequence Read Archive (SRA) under accession numbers SRR33445508 and SRR33445509, as part of the BioProject PRJNA1256155. The associated genome assemblies are accessible under the following BioSample accessions: SAMN48173560 for the T. levigata witches’-broom phytoplasma genome and SAMN48908987 for the T. levigata host genome.

## References

[1] Doi Y, Teranaka M, Yora K, Asuyama H. *Mycoplasma*- or PLT group-like microorganisms found in the phloem elements of plants infected with mulberry dwarf, potato witches’ broom, aster yellows, or paulownia witches’ broom. Jpn J Phytopathol. 1967;33(4):259–66.

[2] Asudi GO, Omenge KM, Paulmann MK, Reichelt M, Grabe V, Mithöfer A, et al. The physiological and biochemical effects on Napier grass plants following Napier grass stunt Phytoplasma infection. Phytopathology. 2021;111(4):703–12. 32997606 10.1094/PHYTO-08-20-0357-R

[3] Huang W, MacLean AM, Sugio A, Maqbool A, Busscher M, Cho S-T, Kamoun S, Kuo C-H, Immink RGH, Hogenhout SA. Parasitic modulation of host development by ubiquitin-independent protein degradation. Cell. 2021;184(20):5201–e52145212. 34536345 10.1016/j.cell.2021.08.029 PMC8525514

[4] Rodrigues Jardim B, Gambley C, Tran-Nguyen LTT, Webster C, Kehoe M, Kinoti WM, Bond S, Davis R, Jones L, Pathania N et al. A metagenomic investigation of Phytoplasma diversity in Australian vegetable growing regions. Microb Genomics. 2024;10(3):001213. 10.1099/mgen.0.001213 PMC10999746 38446015

[5] Wang R, Bai B, Li D, Wang J, Huang W, Wu Y, et al. Phytoplasma: a plant pathogen that cannot be ignored in agricultural production—research progress and outlook. Mol Plant Pathol. 2024;25(2):e13437. 38393681 10.1111/mpp.13437 PMC10887288

[6] Wang X-Y, Zhang R-Y, Li J, Li Y-H, Shan H-L, Li W-F, et al. The diversity, distribution and status of Phytoplasma diseases in China. Front Sustain Food Syst. 2022;Volume 6:2022.

[7] Qiao K, Huang W, Li X, Liang J, Cai H. Combined transcriptomic and metabolomic analyses of defense mechanisms against *Phytoplasma* infection in *Camptotheca acuminata* Decne. Agriculture. 2023. 10.3390/agriculture13101943.

[8] Contaldo N, Satta E, Zambon Y, Paltrinieri S, Bertaccini A. Development and evaluation of different complex media for Phytoplasma isolation and growth. J Microbiol Methods. 2016;127:105–10. 27262375 10.1016/j.mimet.2016.05.031

[9] Wang J, Song L, Jiao Q, Yang S, Gao R, Lu X, et al. Comparative genome analysis of jujube witches’–broom *Phytoplasma*, an obligate pathogen that causes jujube witches’–broom disease. BMC Genomics. 2018;19(1):689. 30231900 10.1186/s12864-018-5075-1 PMC6148798

[10] Zhang R-Y, Wang X-Y, Li J, Shan H-L, Li Y-H, Huang Y-K, He X-H. Complete genome sequence of candidatus Phytoplasma Sacchari obtained using a filter-based DNA enrichment method and nanopore sequencing. Front Microbiol. 2023;14:1252709. 10.3389/fmicb.2023.1252709 PMC10577292 37849920

[11] Debonneville C, Mandelli L, Brodard J, Groux R, Roquis D, Schumpp O. The complete genome of the flavescence dorée phytoplasma reveals characteristics of low genome plasticity. Biology. 2022;11(7):953. 36101334 10.3390/biology11070953 PMC9312162

[12] Nijo T, Iwabuchi N, Tokuda R, Suzuki T, Yamaji Y. Enrichment of phytoplasma genome DNA through a methyl-CpG binding domain-mediated method for efficient genome sequencing. J Gen Plant Pathol. 2021;87(3):1.

[13] Polano C, Firrao G. An effective pipeline based on relative coverage for the genome assembly of phytoplasmas and other fastidious prokaryotes. Curr Genomics. 2018;19(6):491–8. 30258279 10.2174/1389202919666180314114628 PMC6128390

[14] Tokuda R, Iwabuchi N, Kitazawa Y, Nijo T, Suzuki M, Maejima K, Oshima K, Namba S, Yamaji Y. Potential mobile units drive the horizontal transfer of Phytoplasma effector phyllogen genes. Front Genet. 2023; 14:1132432 . 10.3389/fgene.2023.1132432 PMC10210161 37252660

[15] Pacifico D, Galetto L, Rashidi M, Abbà S, Palmano S, Firrao G, Bosco D, Marzachì C, Goodrich-Blair H. Decreasing global transcript levels over time suggest that Phytoplasma cells enter stationary phase during plant and insect colonization. Appl Environ Microbiol. 2015;81(7):2591–602. 25636844 10.1128/AEM.03096-14 PMC4357924

[16] Sugio A, Kingdom HN, MacLean AM, Grieve VM, Hogenhout SA. Phytoplasma protein effector SAP11 enhances insect vector reproduction by manipulating plant development and defense hormone biosynthesis. Proc Natl Acad Sci U S A. 2011;108(48):E1254-63. 22065743 10.1073/pnas.1105664108 PMC3228479

[17] Qionglian W, Lianchun W, Quan W, Xingping X, Jing SFZ. Cai hong: molecular identification of the Phytoplasma associated with Witches’-broom disease in *Trema levigata* and disease survey. Scientia Silvae Sinicae. 2021;57(05):195–201. In Chinese.

[18] Deng S, Hiruki C. Amplification of 16S rRNA genes from culturable and nonculturable mollicutes. J Microbiol Methods. 1991;14(1):53–61.

[19] Schneider B, Seemueller E, Smart CD, Kirkpatrick BC. E6 - Phylogenetic classification of plant pathogenic mycoplasma-like organisms or phytoplasmas. In: Molecular and Diagnostic Procedures in Mycoplasmology*.* Edited by Razin S, Tully JG. San Diego: Academic Press; 1995. p. 369–380.

[20] Gundersen DE, Lee IM. Ultrasensitive detection of phytoplasmas by nested-PCR assays using two universal primer pairs. Phytopathol Mediterr. 1996;35(3):144–51.

[21] Lee IM. Universal amplification and analysis of pathogen 16S rDNA for classification and identification of Mycoplasmalike organisms. Phytopathology. 1993;83(8):834-842.

[22] Aziz RK, Bartels D, Best AA, DeJongh M, Disz T, Edwards RA, et al. The RAST server: rapid annotations using subsystems technology. BMC Genomics. 2008;9:75. 18261238 10.1186/1471-2164-9-75 PMC2265698

[23] Nielsen H, Tsirigos KD, Brunak S, von Heijne G. A brief history of protein sorting prediction. Protein J. 2019;38(3):200–16. 31119599 10.1007/s10930-019-09838-3 PMC6589146

[24] Möller S, Croning MD, Apweiler R. Evaluation of methods for the prediction of membrane spanning regions. Bioinformatics. 2001;17(7):646–53. 11448883 10.1093/bioinformatics/17.7.646

[25] Andersen MT, Liefting LW, Havukkala I, Beever RE. Comparison of the complete genome sequence of two closely related isolates of ‘Candidatus Phytoplasma australiense’ reveals genome plasticity. BMC Genomics. 2013;14:529. 23915186 10.1186/1471-2164-14-529 PMC3750655

[26] Grant JR, Enns E, Marinier E, Mandal A, Herman EK, Chen CY, Graham M, Van Domselaar G, Stothard P. Proksee: in-depth characterization and visualization of bacterial genomes. Nucleic Acids Res. 2023;51(W1):W484–92. 37140037 10.1093/nar/gkad326 PMC10320063

[27] Emms DM, Kelly S. Orthofinder: phylogenetic orthology inference for comparative genomics. Genome Biol. 2019;20(1):238. 31727128 10.1186/s13059-019-1832-y PMC6857279

[28] Edgar RC. Muscle5: High-accuracy alignment ensembles enable unbiased assessments of sequence homology and phylogeny. Nat Commun. 2022;13(1):6968. 36379955 10.1038/s41467-022-34630-w PMC9664440

[29] Talavera G, Castresana J. Improvement of phylogenies after removing divergent and ambiguously aligned blocks from protein sequence alignments. Syst Biol. 2007;56(4):564–77. 17654362 10.1080/10635150701472164

[30] Darriba D, Taboada GL, Doallo R, Posada D. ProtTest 3: fast selection of best-fit models of protein evolution. Bioinformatics. 2011;27(8):1164–5. 21335321 10.1093/bioinformatics/btr088 PMC5215816

[31] Hall BG. Building phylogenetic trees from molecular data with MEGA. Mol Biol Evol. 2013;30(5):1229–35. 23486614 10.1093/molbev/mst012

[32] Kanehisa M, Sato Y, Morishima K. Blastkoala and ghostkoala: KEGG tools for functional characterization of genome and metagenome sequences. J Mol Biol. 2016;428(4):726–31. 26585406 10.1016/j.jmb.2015.11.006

[33] Cantalapiedra CP, Hernández-Plaza A, Letunic I, Bork P, Huerta-Cepas J. eggNOG-mapper v2: functional annotation, orthology assignments, and domain prediction at the metagenomic scale. Mol Biol Evol. 2021;38(12):5825–9. 34597405 10.1093/molbev/msab293 PMC8662613

[34] Monti M, Mandrioli M, Bextine B, Hunter WB, Alma A, Tedeschi R. Maintenance of primary cell cultures of immunocytes from *cacopsylla* spp. psyllids: a new in vitro tool for the study of crop pest insects. In Vitro Cell Dev Biol. 2014;50(9):797–801. 10.1007/s11626-014-9785-7 24934235

[35] Sarhan MS, Patz S, Hamza MA, Youssef HH, Mourad EF, Fayez M, et al. G3 phylochip analysis confirms the promise of plant-based culture media for unlocking the composition and diversity of the maize root microbiome and for recovering unculturable candidate divisions/phyla. Microbes Environ. 2018;33(3):317–25. 30210099 10.1264/jsme2.ME18023 PMC6167109

[36] Tanno K, Maejima K, Miyazaki A, Koinuma H, Iwabuchi N, Kitazawa Y, et al. Comprehensive screening of antimicrobials to control *Phytoplasma* diseases using an in vitro plant–*phytoplasma* co-culture system. Microbiology. 2018;164(8):1048–58. 29952745 10.1099/mic.0.000681

[37] Chen W, Li Y, Wang Q, Wang N, Wu Y. Comparative genome analysis of wheat blue dwarf Phytoplasma, an obligate pathogen that causes wheat blue dwarf disease in China. PLoS ONE. 2014;9(5):e96436. 24798075 10.1371/journal.pone.0096436 PMC4010473

[38] Kube M, Schneider B, Kuhl H, Dandekar T, Heitmann K, Migdoll AM, et al. The linear chromosome of the plant-pathogenic Mycoplasma ‘Candidatus Phytoplasma *mali*.’ BMC Genomics. 2008;9(1):306. 18582369 10.1186/1471-2164-9-306 PMC2459194

[39] Kube M, Mitrovic J, Duduk B, Rabus R, Seemüller E. Current view on Phytoplasma genomes and encoded metabolism. Sci World J. 2012. 10.1100/2012/185942. 10.1100/2012/185942 PMC3322544 22550465

[40] Baric S, Berger J, Cainelli C, Kerschbamer C, Dalla Via J. Molecular typing of ‘Candidatus Phytoplasma mali’ and epidemic history tracing by a combined T-RFLP/VNTR analysis approach. Eur J Plant Pathol. 2011;131(4):573–84.

[41] Huang C-T, Cho S-T, Lin Y-C, Tan C-M, Chiu Y-C, Yang J-Y, Kuo C-H. Comparative genome analysis of ‘Candidatus Phytoplasma luffae’ reveals the influential roles of potential mobile units in Phytoplasma evolution. Front Microbiol. 2022;13:773608. 10.3389/fmicb.2022.773608 PMC8923039 35300489

[42] Zheng Y, Li J, Zheng M, Li Y, Deng X, Zheng Z. Whole genome sequences of 135 *candidatus liberibacter* asiaticus strains from China. Sci Data. 2024;11(1):1018. 39300139 10.1038/s41597-024-03855-3 PMC11413205

[43] Zhao Y, Wei W, Davis RE, Lee I-M, Bottner-Parker KD. The agent associated with blue dwarf disease in wheat represents a new phytoplasma taxon, ‘Candidatus Phytoplasma tritici’. Int J Syst Evol MicroBiol. 2021;71:004604. 10.1099/ijsem.0.004604 33464199

[44] Li J, Chen L, Wang H, Chen P, Yang Q, Zhang Y, et al. Research progress on the pathogenic mechanism and control of jujube witches’broom disease. J Henan Agric Univ. 2021;55(1):1–714.

[45] Kämpfer P, Glaeser SP. Prokaryotic taxonomy in the sequencing era – the polyphasic approach revisited. Environ Microbiol. 2012;14(2):291–317. 22040009 10.1111/j.1462-2920.2011.02615.x

[46] Bertaccini A, Arocha-Rosete Y, Contaldo N, Duduk B, Fiore N, Montano HG, Kube M, Kuo C-H, Martini M, Oshima K et al. Revision of the ‘Candidatus phytoplasma’ species description guidelines. Int J Syst Evol MicroBiol. 2022;72:005353 . 10.1099/ijsem.0.005353 35471141

[47] Siewert C, Luge T, Duduk B, Seemüller E, Büttner C, Sauer S, et al. Analysis of expressed genes of the bacterium ‘Candidatus Phytoplasma mali’ highlights key features of virulence and metabolism. PLoS ONE. 2014;9(4):e94391. 24728201 10.1371/journal.pone.0094391 PMC3984173

[48] Fernández FD, Yan X-H, Kuo C-H, Marcone C, Conci LR. Improving the comprehension of pathogenicity and phylogeny in ‘Candidatus Phytoplasma meliae’ through genome characterization. Microorganisms. 2024;12(1):142. 38257969 10.3390/microorganisms12010142 PMC10819327

[49] Economou A. Following the leader: bacterial protein export through the sec pathway. Trends Microbiol. 1999;7(8):315–20. 10431204 10.1016/s0966-842x(99)01555-3

[50] Tjalsma H, Bolhuis A, Jongbloed Jan DH, Bron S, van Dijl Jan M. Signal Peptide-Dependent protein transport InBacillus subtilis: a Genome-Based survey of the secretome. Microbiol Mol Biol Rev. 2000;64(3):515–47. 10974125 10.1128/mmbr.64.3.515-547.2000 PMC99003

[51] Oshima K, Maejima K, Namba S. Genomic and evolutionary aspects of phytoplasmas. Front Microbiol. 2013;4:230. 23966988 10.3389/fmicb.2013.00230 PMC3743221

[52] Kakizawa S, Oshima, Kenro N, Wei HH-YJ. Secretion of immunodominant membrane protein from onion yellows Phytoplasma through the sec protein-translocation system in Escherichia coli. Microbiology. 2004;150(1):135–42. 14702406 10.1099/mic.0.26521-0

[53] Wei W, Kakizawa S, Jung H-Y, Suzuki S, Tanaka M, Nishigawa H, et al. An antibody against the SecA membrane protein of one Phytoplasma reacts with those of phylogenetically different phytoplasmas. Phytopathology®. 2004;94(7):683–6. 18943899 10.1094/PHYTO.2004.94.7.683

[54] Bai X, Zhang J, Ewing A, Miller SA, Radek AJ, Shevchenko DV, et al. Living with genome instability: the adaptation of phytoplasmas todiverse environments of their insect and plant hosts. J Bacteriol. 2006;188(10):3682–96. 16672622 10.1128/JB.188.10.3682-3696.2006 PMC1482866

[55] Tran-Nguyen LT, Kube M, Schneider B, Reinhardt R, Gibb KS. Comparative genome analysis of *candidatus* Phytoplasma australiense (subgroup tuf-Australia I; rp-A) and Ca. Phytoplasma asteris strains OY-M and AY-WB. J Bacteriol. 2008;190(11):3979–91. 18359806 10.1128/JB.01301-07 PMC2395047

[56] Liu T, Qu J, Fang Y, Yang H, Lai W, Pan L, et al. Polyamines: the valuable bio-stimulants and endogenous signaling molecules for plant development and stress response. J Integr Plant Biol. 2025;67(3):582–95. 39601632 10.1111/jipb.13796

[57] Majumdar R, Minocha R, Lebar MD, Rajasekaran K, Long S, Carter-Wientjes C, Minocha S, Cary JW. Contribution of maize polyamine and amino acid metabolism toward resistance against Aspergillus flavus infection and aflatoxin production. Front Plant Sci. 2019;10. 10.3389/fpls.2019.00692 PMC6543017 31178889

[58] Sun Q, Hao Y, Liu Y, Cui M, Zhang G, Yu W, et al. Identification and characterization of polyamine metabolism in citrus in response to ‘Candidatus liberibacter asiaticus’ infection. Phytopathology. 2024;114(6):1380–92. 38349804 10.1094/PHYTO-04-23-0114-R

[59] Plett JM, Plett KL, Wong-Bajracharya J, de Freitas Pereira M, Costa MD, Kohler A, et al. Mycorrhizal effector PaMiSSP10b alters polyamine biosynthesis in Eucalyptus root cells and promotes root colonization. New Phytol. 2020;228(2):712–27. 32562507 10.1111/nph.16759

[60] Oshima K, Kakizawa S, Nishigawa H, Jung HY, Wei W, Suzuki S, et al. Reductive evolution suggested from the complete genome sequence of a plant-pathogenic phytoplasma. Nat Genet. 2004;36(1):27–9. 14661021 10.1038/ng1277

[61] Wei W, Trivellone V, Dietrich CH, Zhao Y, Bottner-Parker KD, Ivanauskas A. Identification of phytoplasmas representing multiple new genetic lineages from phloem-feeding leafhoppers highlights the diversity of phytoplasmas and their potential vectors. Pathogens. 2021;10(3):352. 33809759 10.3390/pathogens10030352 PMC8002289

[62] Janik K, Mithöfer A, Raffeiner M, Stellmach H, Hause B, Schlink K. An effector of Apple proliferation Phytoplasma targets TCP transcription factorsa generalized virulence strategy of Phytoplasma? Mol Plant Pathol. 2017;18(3):435–42. 27037957 10.1111/mpp.12409 PMC6638208

[63] Zhang LY, Du YX, Qu Q, Zheng QY. Structure basis for recognition of plant Rpn10 by Phytoplasma SAP05 in ubiquitin-independent protein degradation. iScience. 2024. 10.1016/j.isci.2024.108892. 38322988 10.1016/j.isci.2024.108892 PMC10844826

[64] Fernández FD, Debat HJ, Conci LR. Molecular characterization of effector protein SAP54 in bellis virescence Phytoplasma (16SrIII-J). Trop Plant Pathol. 2019;44(4):392–7.

[65] Hemmati C, Nikooei M, Al-Subhi AM, Al-Sadi AM. History and current status of phytoplasma diseases in the Middle East. Biology. 2021. 10.3390/biology10030226. 33804178 10.3390/biology10030226 PMC8000475

[66] Liao P-Q, Chen Y-K, Mejia HM, Chien Y-Y, Lee Y-C, Tan C-M, et al. Detection, identification, and molecular characterization of a 16SrII-V subgroup Phytoplasma associated with *Nicotiana plumbaginifolia*. Plant Dis. 2022;106(3):805–9. 34763517 10.1094/PDIS-09-21-1968-SC

[67] Aurin MB, Haupt M, Görlach M, Rümpler F, Theissen G. Structural requirements of the Phytoplasma effector protein SAP54 for causing homeotic transformation of floral organs. Mol Plant Microbe Interact. 2020;33(9):1129–41. 32689871 10.1094/MPMI-02-20-0028-R

[68] Xue C, Zhang Y, Li H, Liu Z, Gao W, Liu M, et al. The genome of candidatus *Phytoplasma ziziphi* provides insights into their biological characteristics. BMC Plant Biol. 2023;23(1):251. 37173622 10.1186/s12870-023-04243-6 PMC10176825

[69] Wei W, Inaba J, Zhao Y, Mowery JD, Hammond R. Phytoplasma infection blocks starch breakdown and triggers chloroplast degradation, leading to premature leaf senescence, sucrose reallocation, and spatiotemporal redistribution of phytohormones. Int J Mol Sci. 2022. 10.3390/ijms23031810. 35163732 10.3390/ijms23031810 PMC8836287

