# Supplementary material for: Genomic insights into the pathogenicity of a ‘Candidatus Phytoplasma asteris’ associated with Trema levigata witches’ broom disease in China
Source: BMC Plant Biol. 2025 Oct 28;25:1459. doi: 10.1186/s12870-025-07482-x (PMC12570602; doi:10.1186/s12870-025-07482-x)
Supplement: Supplementary file 2 — Supplementary Material 2. Additional file S2: Supplementary figure S2. Supplemental Fig. 1: Original agarose gel image of PCR detection for phytoplasma; Supplemental Fig. 2: Field symptoms of witches’ broom disease on Trema levigata [file 12870_2025_7482_MOESM2_ESM.docx]

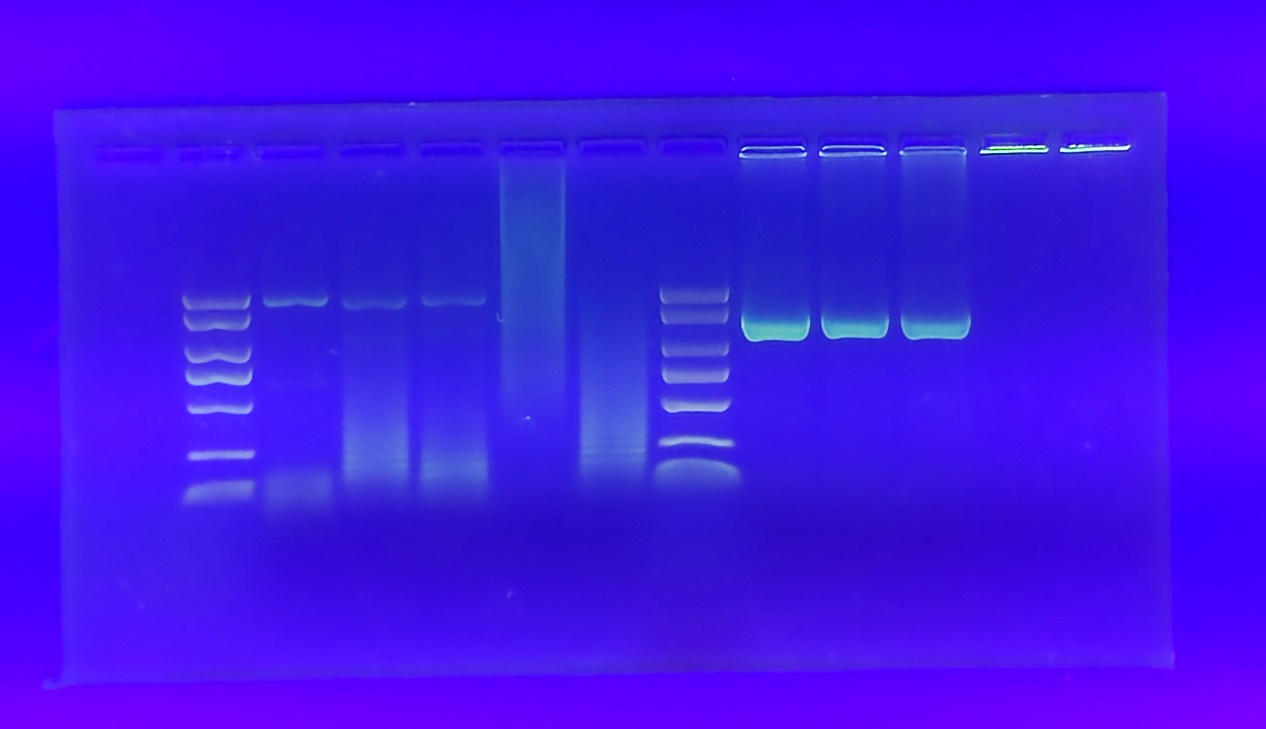


**Supplemental Figure 1:** **Original agarose gel image of PCR detection for phytoplasma**


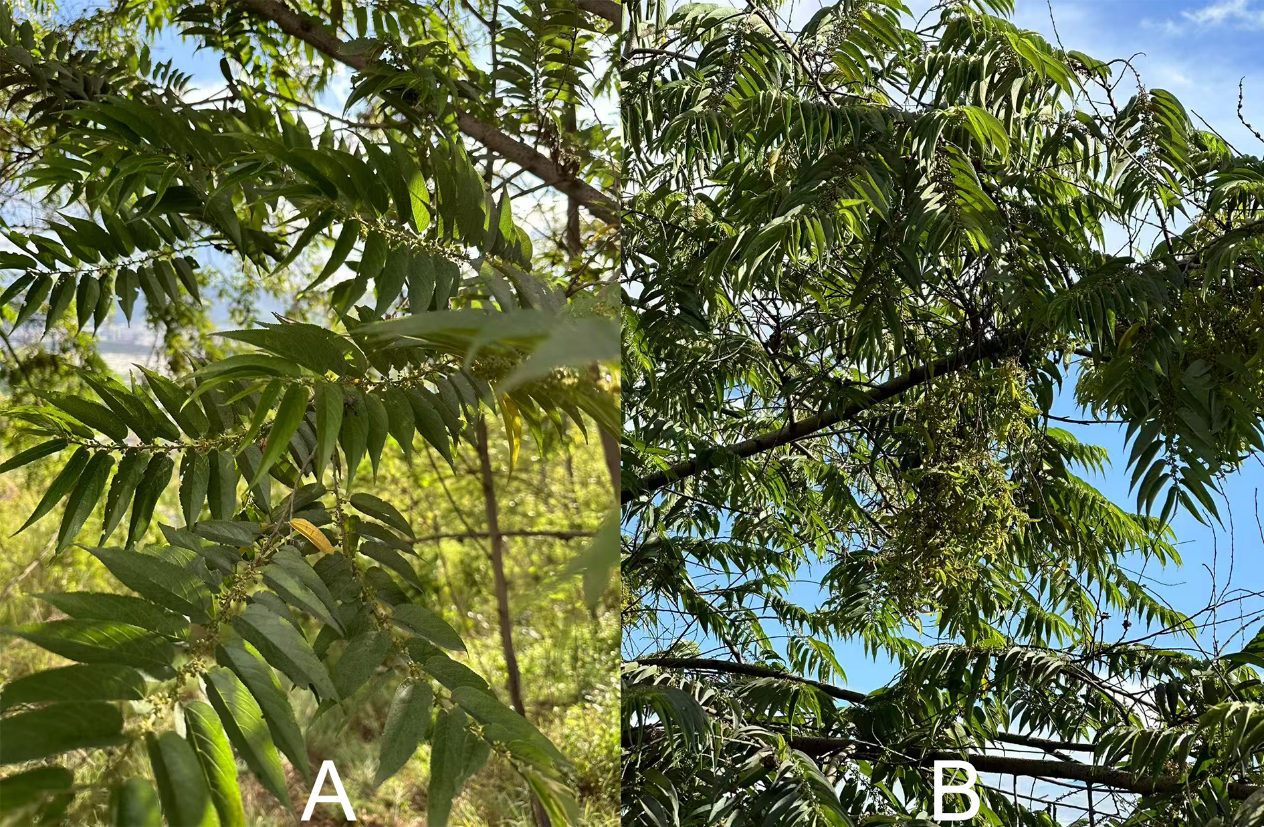


**Supplemental Figure 2:** **Field symptoms of** **witches' broom disease on*Trema levigata***

**A:Health ；B：witches' broom disease on *Trema levigata***
